# Supplementary material for: Complexome profiling of the Chlamydomonas psb28 mutant reveals TEF5 as an early PSII assembly factor
Source: Plant Cell. 2025 Mar 17;37(6):koaf055. doi: 10.1093/plcell/koaf055 (PMC12164590; doi:10.1093/plcell/koaf055)
Supplement: koaf055_Supplementary_Data [file koaf055_supplementary_data.zip › psb28_interactive.zip!/plots/intensity/qualityPlots/peptides/ALD1_Cre12.g520350.t1.2.html]

### Cre12.g520350.t1.2

Trivial name: ALD1  
SeqCoverage [median]: 3.60  
Mass: 59.25 kDa  
  
 

| Sequence | MissedCleavage | Mass [Da] | wtIntensity | muIntensity | Score |
| --- | --- | --- | --- | --- | --- |
| SYDQALGEVR | 0 | 1136.55 | 0.00 | 1268.66 | 99.80 |
| VSVVAEQLVR | 0 | 1098.64 | 0.00 | 3039.58 | 64.04 |
|  |  |  | 0.0 | 4308.2 |  |
